# The Arabidopsis RLCK VI_A2 Kinase Controls Seedling and Plant Growth in Parallel with Gibberellin

**DOI:** 10.3390/ijms21197266

**Published:** 2020-10-01

**Authors:** Ildikó Valkai, Erzsébet Kénesi, Ildikó Domonkos, Ferhan Ayaydin, Danuše Tarkowská, Miroslav Strnad, Anikó Faragó, László Bodai, Attila Fehér

**Affiliations:** 1 Institute of Plant Biology, Biological Research Centre, 6726 Szeged, Hungary; ildiko.valkai@gmail.com (I.V.); kenesi.erzsebet@brc.hu (E.K.); domonkos.ildiko@brc.hu (I.D.); ayaydin.ferhan@brc.hu (F.A.); 2 Laboratory of Growth Regulators, Institute of Experimental Botany, Czech Academy of Sciences and Palacky University, 78371 Olomouc, Czech Republic; tarkowska@ueb.cas.cz (D.T.); miroslav.strnad@upol.cz (M.S.); 3 Department of Biochemistry and Molecular Biology, Faculty of Science and Informatics, University of Szeged, 6726 Szeged, Hungary; f.ancsi93@freemail.hu (A.F.); bodai@bio.u-szeged.hu (L.B.); 4 Doctoral School in Biology, Faculty of Science and Informatics, University of Szeged, 6726 Szeged, Hungary; 5 Department of Plant Biology, Faculty of Science and Informatics, University of Szeged, 6726 Szeged, Hungary

**Keywords:** *Arabidopsis thaliana*, cell expansion, gibberellins, hypocotyl growth, transcriptomic analysis, plant hormones, plant size, receptor-like cytoplasmic kinase, skotomorphogenesis

## Abstract

The plant-specific receptor-like cytoplasmic kinases (RLCKs) form a large, poorly characterized family. Members of the RLCK VI_A class of dicots have a unique characteristic: their activity is regulated by Rho-of-plants (ROP) GTPases. The biological function of one of these kinases was investigated using a T-DNA insertion mutant and RNA interference. Loss of RLCK VI_A2 function resulted in restricted cell expansion and seedling growth. Although these phenotypes could be rescued by exogenous gibberellin, the mutant did not exhibit lower levels of active gibberellins nor decreased gibberellin sensitivity. Transcriptome analysis confirmed that gibberellin is not the direct target of the kinase; its absence rather affected the metabolism and signalling of other hormones such as auxin. It is hypothesized that gibberellins and the RLCK VI_A2 kinase act in parallel to regulate cell expansion and plant growth. Gene expression studies also indicated that the kinase might have an overlapping role with the transcription factor circuit (PIF4-BZR1-ARF6) controlling skotomorphogenesis-related hypocotyl/cotyledon elongation. Furthermore, the transcriptomic changes revealed that the loss of *RLCK VI_A2* function alters cellular processes that are associated with cell membranes, take place at the cell periphery or in the apoplast, and are related to cellular transport and/or cell wall reorganisation.

## 1. Introduction

Eukaryotic protein kinases form a large superfamily, and are associated essentially with all cellular functions. During evolution, protein kinase families have evolved independently in the lineages of eukaryotes. In consequence, the various kinase families are unevenly represented in the different eukaryotic organisms, which also have specific kinase classes. The number of protein kinase coding genes is especially high in plant genomes. In *Arabidopsis thaliana*, ~4% of the protein coding genes code for protein kinases while this percentage is ca. 2% in *Homo sapiens* [1]. This high number of protein kinases is likely due to the importance of cell-to-cell communication during the post-embryonic development of plants that is strongly influenced by the environment. In addition, plant defence and immunity depend on the specific recognition of pathogen-associated molecular patterns. In plants, cell-to-cell communication as well as innate immunity rely on plant receptor-like kinases (RLKs), which account for more than half of protein kinases in Arabidopsis (>600 RLK genes out of >1000 kinase-coding genes). RLKs resemble the receptor kinases of animals. The sequence of their kinase domain indicates that they are rather related to the animal cytoplasmic Pelle and interleukin receptor-associated kinases [2]. Moreover, RLKs exhibit serine/threonine kinase specificity in contrast to animal receptor kinases that are almost exclusively tyrosine kinases. This indicates that ancient RLK/Pelle kinases were co-opted for transmembrane signalling in plants after their divergence from animals.

RLKs can be classified into several families based on their various extracellular ligand-binding and slightly divergent cytoplasmic kinase domains [2]. There are also a number of RLK-like kinases that have only cytoplasmic kinase domain but no extracellular ligand-binding domain (only a few of them have a transmembrane domain) [3]. These cytoplasmic protein kinases are referred to as receptor-like cytoplasmic kinases, or receptor-like cytoplasmic kinases (RLCKs). The 149 RLCKs of Arabidopsis were divided into 17 subfamilies (RLCK-II and RLCK-IV to RLCK-XIX), based on sequence homology [3]. RLCKs often associate with RLKs to mediate cellular signalling in response to various RLK-sensed environmental and/or developmental signals [4]. Most of the RLCKs, however, have unknown functions.

The Arabidopsis RLCK-VI family is divided into two groups, with seven members each: RLCK VI_A and RLCK VI_B [5]. RLCK VI_A but not RLCK VI_B kinases were shown to bind plant Rho-type small GTPases (ROPs) in their GTP-bound state [6,7,8,9]. This binding results in augmented in vitro kinase activity [6,8,9]. Regulation of kinase activity by Rho-type GTPases is well known in animal and yeast cells as well. In these organisms, the kinase classes regulated by Rho-type Rho/Rac/Cdc42 GTPases are the p21-activated kinases (PAKs), the Rho-kinases (ROKs), the mixed-lineage kinases (MLKs), the myotonin-related Cdc42-binding kinases (MRCKs), the citron kinases (CRIKs), and the novel protein kinase (PKN) [10]. However, plant genomes code for none of these kinases [11]. It seems that, during the evolution of land plants, a sub-group of plant-specific RLCKs were co-opted to mediate ROP GTPase signalling [12]. While the GTPase-binding ability of yeast and animal Rho-type GTPase-regulated kinases is due to the presence of defined structural elements outside of their kinase domains (such as the Cdc42/Rac-interactive binding -CRIB- motif of PAKs), RLCK VI_A kinases use conserved amino acids widely distributed in the kinase domain to form a binding surface for ROPs [12].

At present, the members of the RLCK VI_A group are the only known plant kinases for which the activity is directly regulated by ROP GTPases, at least in vitro [6,8,9]. This fact is rather surprising, considering the wide role of animal Rho-type GTPases in kinase signalling [10], as well as the central role of ROP GTPases in a variety of cellular functions [13]. Despite their unique regulation, the biological function of RLCK VI_A kinases has hardly been investigated so far [11]. The barley HvRBK1 kinase (homologue of the Arabidopsis RLCK VI_A3 kinase) was shown to have a role in basal disease resistance [6]. Transient silencing of the gene decreased the stability of cortical microtubules and promoted fungal penetration into barley epidermal cells. Mutation in the gene coding for the Arabidopsis homologue of HvRBK1, AtRLCK VI_A3 was reported to support fungal reproduction [9]. Arabidopsis *AtRBK1* (*RLCK VI_A4*) and *AtRBK2* (*RLCK VI_A6*) genes were shown to have augmented expression following pathogen infection, supporting the general role of RLCK VI_A kinases in pathogen responses. The *atrlck vi_a3* mutant also exhibited reduced plant size and an increase in the ratio of trichomes with high branch numbers, while the AtRBK1 (RLCK VI_A4) kinase was found to be a member of a kinase cascade regulating auxin-mediated cell elongation, consistent with a developmental/morphogenic role [14].

Here, we report the involvement of the *AtRLCK VI_A2* gene in the regulation of plant growth and (skoto)morphogenesis. T-DNA insertion into the gene resulted in reduced hypocotyl elongation and smaller rosette size, which could be ascribed to limited cell expansion. These mutant phenotypes were complemented by the exogenous application of gibberellic acid. Measurements could not reveal differences neither in the gibberellin content nor in the gibberellin sensitivity of the mutant. Transcript analysis indicated that the kinase might indirectly affect gibberellin-dependent responses during skotomorphogensis, by overlapping with the action of the central transcription factor network regulating hypocotyl growth, and interfering with hormone signalling, cell wall organisation, and cellular transport processes.

## 2. Results

### 2.1. Molecular Characterization of the RLCK VI_A2 T-DNA Insertion Mutant and the Transgenic Plants Used in the Study

**Figure 1:**
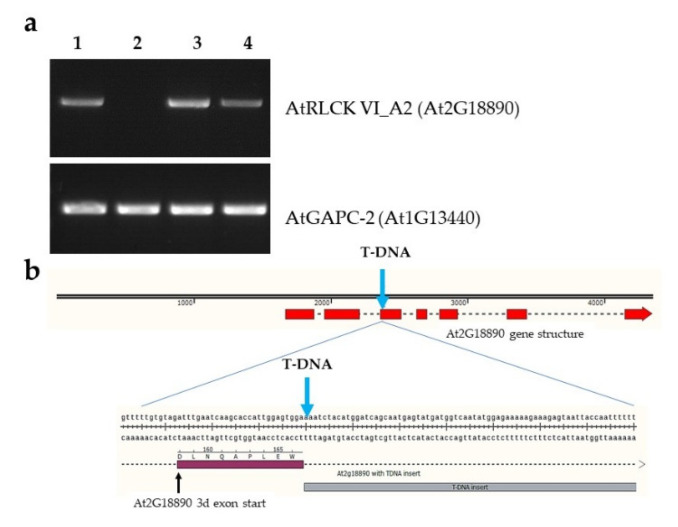
Expression of the receptor-like cytoplasmic kinase (*RLCK*) *VI_A2* gene in various Arabidopsis lines used in the study. (**a**) RT-PCR results using *RLCK VI_A2* (upper row) and *GAPC-2* specific (lower row) primers in wild type (**1**), T-DNA insertion line GABI_435H03 (**2**), T-DNA insertion line GABI_676D12 (**3**), and the GABI_435H03 line expressing the *RLCK VI_A2* cDNA transgene under the control of the 35S promoter (complemented mutant) (**4**). (**b**) Site of the T-DNA insertion in the third exon of the At2G18890 gene coding for the AtRLCK VI_A2 kinase in the GABI_435H03 line.

In order to reveal possible biological functions of the ROP GTP-ase binding AtRLCK VI_A2 (At2G18890) kinase [8], we carried out a search in the GABI-Kat Arabidopsis T-DNA insertional mutant collection [15]. Two lines with predicted T-DNA insertion in the 5’ untranslated region (GABI_676D12) or in the second intron of the At2G18890 gene (GABI_435H03), respectively, could be identified. Seeds of the T-DNA mutants were obtained from the Nottingham Arabidopsis Stock Centre (NASC) [16]. Expression of the At2G18890 gene was tested in the homozygous lines using a specific PCR primer pair, amplifying the whole transcript by RT-PCR. It could be established that the GABI_435H03 line does not produce *RLCK VI_A2* transcripts in contrast to the line GABI_676D12 (Figure 1a). In order to determine the exact T-DNA insertion site, the junction region of the At2G18890 gene and the T-DNA was amplified from the genomic DNA of the GABI_435H03 line using a T-DNA-specific reverse primer and an At2G18890 second exon specific forward PCR primer. Sequencing of the PCR product verified that the T-DNA insertion was located not in the second intron but in the third exon (at the ninth codon after the intron/exon junction) (Figure 1b). Mapping transcript reads of the *rlck vi_a2* mutant by next generation sequencing (NGS) to the reference *Arabidopsis thaliana* genome confirmed that, although the first two exons are transcribed in the mutant, full length functional transcripts are not produced and therefore the mutant can be considered as a knock out (Supplementary Figure S1).

To validate the mutant phenotypes, various transgenic Arabidopsis lines were produced. The *rlck vi_a2* mutant was complemented with 35S-promoter-driven expression of the At2G18890 cDNA N-terminally fused, with a TAP-tag that allows co-immunoprecipitation of kinase interacting proteins [17]. Based on gene expression verification, a representative line was selected for further studies (Figure 1a). Furthermore, estradiol-induced RNA-interference [18] was used to knock down *RLCK VI_A2* expression (Supplementary Figure S2a) to further verify some of the experimental findings obtained with the mutant line.

### 2.2. The RLCK VI_A2 Kinase Controls Seedling and Plant Growth

**Figure 2:**
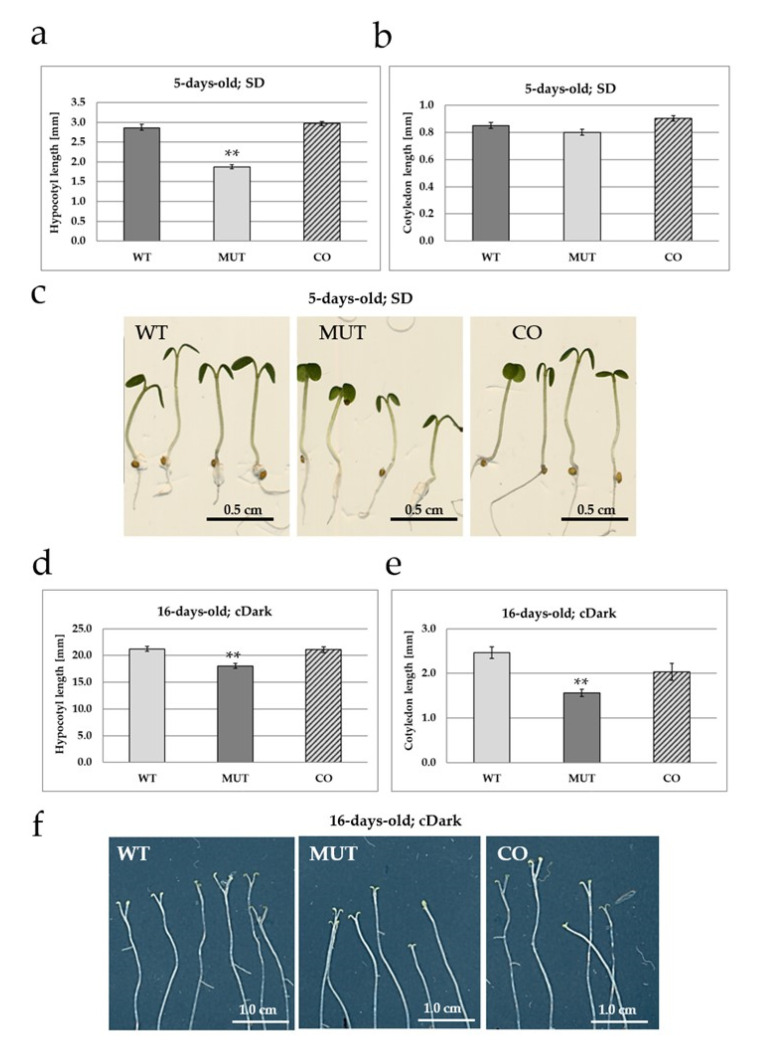
The *rlck vi_a2* mutation affects hypocotyl and cotyledon elongation. Hypocotyl (**a**,**d**) and cotyledon (**b**,**e**) length were measured for 5-days-old short-day (SD; 8/16h light/dark cycle) and 16-days-old dark-grown (cDark; continuous dark) seedlings. WT—wild type; MUT—T-DNA insertion mutant line; CO—complemented mutant line. Three biological replicates were made with 15–25 plants per line. Averages and standard errors are shown. Corresponding representative images are displayed on (**c**,**f**). ** *p* < 0.005 (Student’s *t*-test; comparison to WT).

The *rlck vi_a2* mutant seedlings that were grown under 8 h/16 h light/dark periods for 5 days exhibited significantly shorter hypocotyls as compared to the wild type (Figure 2a,c). Ectopic expression of the kinase in the mutant background restored hypocotyl growth to normal (Figure 2a,c). The cotyledons were also smaller in the mutant, although this difference was not statistically significant (Figure 2b). Since hypocotyl growth is accelerated in the dark, seeds were also germinated and cultured under continuous darkness for 16 days, and the size of the hypocotyls and cotyledons was compared (Figure 2d–f).

The hypocotyl of the mutant was found to be significantly shorter under this condition as well, while the wild type and complemented lines exhibited similar hypocotyl sizes (Figure 2d,f). The length of cotyledons, especially that of their petioles, were significantly reduced in the mutant but was restored to the wild type level in the complemented line (Figure 2e,f). The phenotypes of mutant seedlings could be recreated via estradiol-induced silencing of the *RLCK VI_A2* gene in transgenic seedlings (Supplementary Figure S2b).

**Figure 3:**
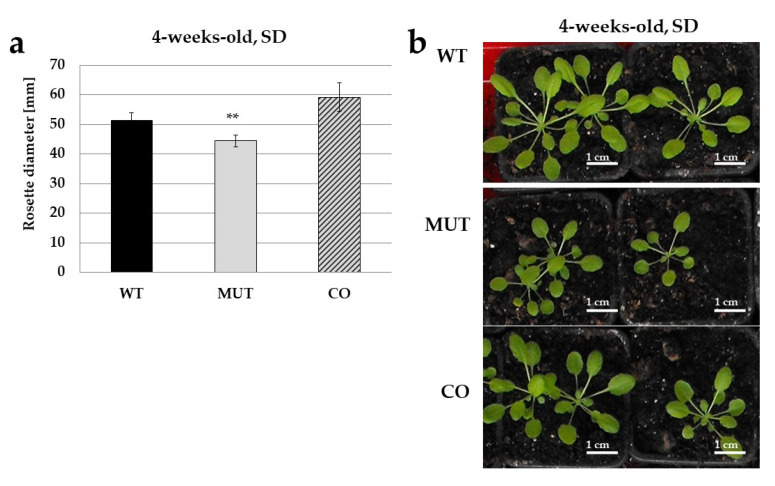
Greenhouse-grown mutant (MUT) plants exhibited smaller plant size, as evidenced by measuring the rosette diameter. Normal size of the wild type (WT) plants was restored by expressing the kinase cDNA in the mutant background (complemented, CO line). Rosette diameters in mm are shown in (**a**), and representative images of the measured 4-weeks-old plants in (**b**). The plants were grown in short day conditions (SD). Averages and standard errors were calculated and are shown on (**a**). *n* = 15–25, ** *p* < 0.005 (Student’s *t*-test; comparison to WT).

Seedlings were also grown into plants in pots in the greenhouse under short-day condition (8 h light, 16 h dark). A significant difference in the size of the rosettes was observed: it was decreased in the mutant, but restored to the normal level in the complemented transgenic line as compared to the wild type control (Figure 3).

Altogether, these data indicate that the RLCK VI_A2 kinase is required for normal plant growth under light as well as in dark; in seedlings as well as in greenhouse plants.

### 2.3. The RLCK VI_A2 Kinase Controls Cell Size

**Figure 4:**
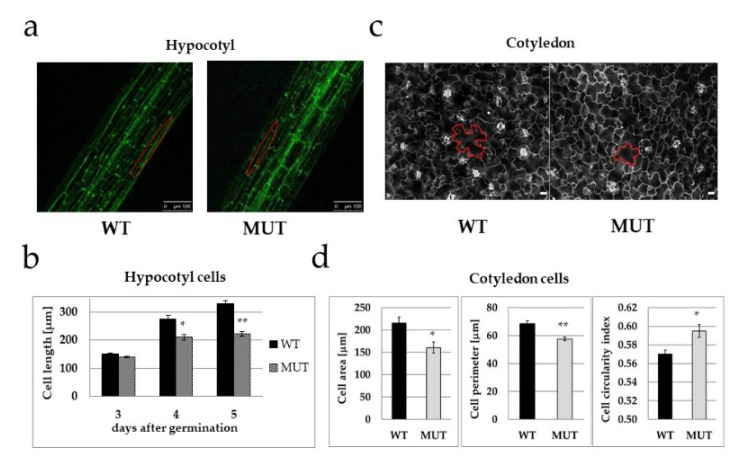
The sizes of hypocotyl (**a**,**b**) and cotyledon (**c**,**d**) cells are significantly smaller in the mutant (MUT) than in the wild type (WT) plants. Fluorescent (**a**) and scanning electron microscopic (**c**) images are shown for the epidermal cells of the hypocotyl (**a**) and the cotyledon (**c**), respectively, of 5-day-old seedlings. The white bars indicate 100 μm (**a**), and 10 μm (**b**), respectively. For the quantitative comparison of cell size (**b**,**d**), 150–200 cells were measured for each of three randomly selected seedlings per line. Averages and standard errors are shown on the histograms. * *p* < 0.05; ** *p* < 0.005 (Student’s *t*-test; comparison to WT).

Microscopic investigations revealed that epidermal cell size was significantly smaller in both investigated organs of the mutant seedlings (Figure 4). In the mutant, hypocotyl epidermal cells were less elongated (Figure 4a,b), while the epidermal cells of the cotyledon were not only smaller in area, but their shape was also different; their circularity index was much higher (Figure 4c,d), indicating limited planar polarity [19].

### 2.4. Gibberellic Acid Treatment Rectifies the rlck vi_a2 Mutant Phenotypes

**Figure 5:**
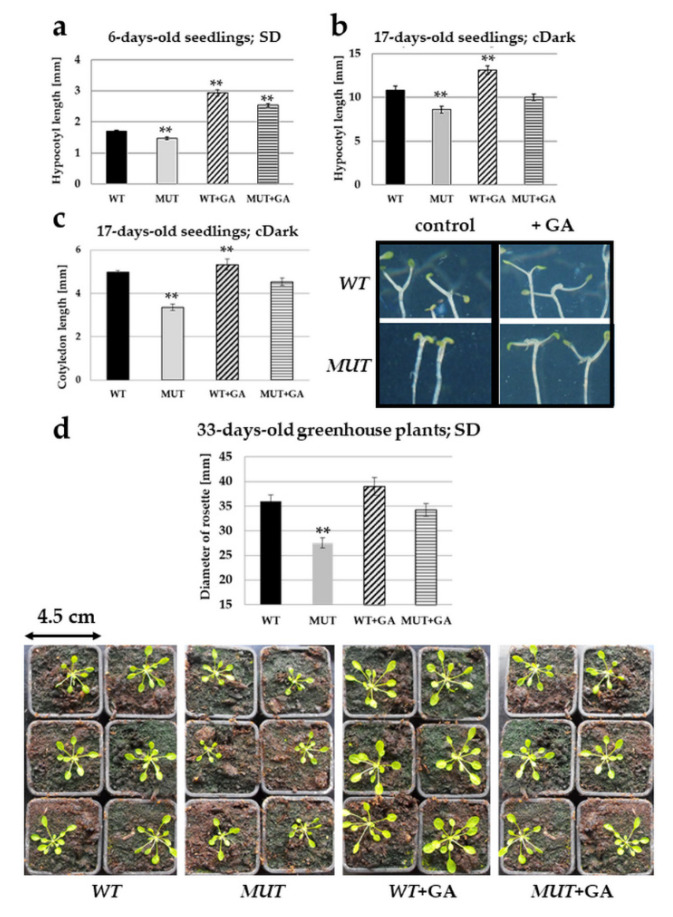
Exogenous gibberellin treatments complemented the mutant phenotypes. Wild type (WT) or *rlck vi_a2* mutant (MUT) seedlings grown in vitro in short days (SD; 8 h/16 h light/dark) for 6 days (**a**) or in continuous dark (cDark) for 17 days (**b**,**c**) and plants grown at short days in greenhouse (**d**) were or were not treated with 20 μM gibberellic acid (GA_3_). Hypocotyl length (**a**,**b**), cotyledon length (**c**) or rosette diameter (**d**) were measured in 15–25 seedlings or plants, respectively, in three repetitions. Averages and standard errors are shown. ** *p* < 0.05 (Student’s *t*-test; comparison to WT).

In order to test whether the mutant phenotype can be linked to the disturbed action of plant hormones, seedlings were grown in the presence of various plant growth regulators (5 nM indoleacetic acid, 1 μM brassinolide, 100 μM ethephon (Ethrel) or 20 μM gibberellic acid (GA_3_)) and hypocotyl, cotyledon and rosette sizes were measured (Supplementary Figures S3 and S4). Of the investigated hormones, exogenous gibberellin (GA_3_) was found to rectify the growth defects of the *rlck vi_a2* mutant (Figure 5) and the RNAi-silenced lines (Supplementary Figure S4).

### 2.5. The Effect of the rlck vi_a2 Mutation on Gibberellic Acid Level, Synthesis, and Signalling in Seedlings

**Figure 6:**
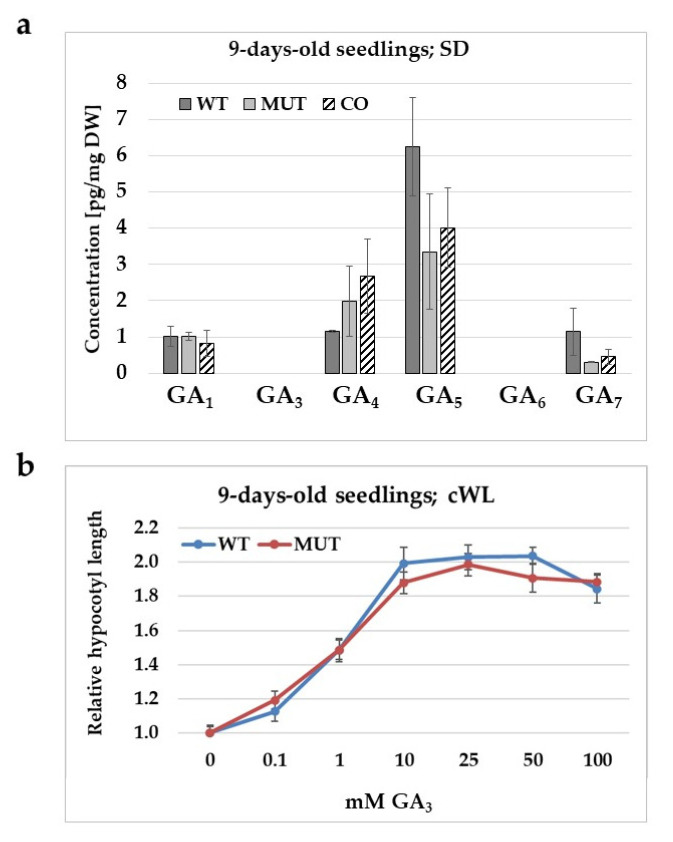
Gibberellin content and gibberellin sensitivity of the mutant and the wild type. (**a**) Endogenous content of active gibberellins was measured in 9-days-old seedlings of wild type (WT), *rlck vi_a2* mutant (MUT), and complemented mutant (CO) lines. Seedlings were grown in vitro under short day (SD; 8 h/16 h light/dark) conditions in a growth chamber. Samples were collected from three independent experiments. Averaged data are shown with the standard deviations. No statistically significant differences could be observed among the tested Arabidopsis lines (*p* < 0.05 Student’s *t*-test; comparison to WT). (**b**) Relative hypocotyl length was determined in response to a range of GA_3_ concentrations (0–100 μM), in the case of wild type and mutant seedlings (9-days-old; grown under low intensity continuous white light).

Endogenous level of gibberellins (GAs) having biological activity was determined in the case of wild type, and mutant and complemented mutant seedlings (Figure 6a). The major bioactive GAs include GA_1_, GA_3_, GA_4_, and GA_7_, but GA_5_ and GA_6_ have also been indicated to have biological activities [20]. In 9-day-old seedlings, among these GAs, GA_5_ exhibited the highest concentration, GA_1_, GA_4_ and GA_7_ was found to have lower levels, while GA_3_ and GA_6_ could not be detected. No significant differences were found in the concentration of active GAs in the seedlings of the various Arabidopsis lines tested. The GA sensitivity of the mutant was also compared to that of the wild type control. It was found that hypocotyl growth was not less responsive to exogenous GA_3_ in the mutant than in the wild type (Figure 6b).

### 2.6. Transcriptome Analysis Indicated a Gibberellin-Independent Role for the RLCK VI_A2 Kinase in Skotomorphogenesis

To clarify the role of the RLCK VI_A2 kinase in seedling growth and cell elongation, the transcriptome of 18-days-old dark-grown wild-type and *rlck vi_a2* mutant seedlings were compared in three biological replicates. 1203 statistically highly significant (*q* < 0.05; fold change > 1.5) DEGs (differentially expressed genes) were identified, among which 406 were upregulated and 707 downregulated (Supplementary Table S1). Gene ontology (GO) enrichment analysis (Plant GOSlim ontologies) indicated the involvement of the kinase in the responses to various exogenous and endogenous stimuli including biotic and abiotic stresses and plant hormones as well as in lipid, carbohydrate, and secondary metabolism (Table 1). A more detailed GO analysis (complete GO ontologies) showed that the kinase might modulate the signalling, metabolism and transport of several hormones, including auxin, abscisic acid, jasmonic acid, and salicylic acid, but gibberellic acid-related DEGs were not significantly enriched in the mutant (Supplementary Tables S2 and S3).

Browsing the annotation of those DEGs that have the GO “hormone response” for the key word “gibberellin” revealed a small number of GA-regulated proteins (Table 2 and Supplementary Table S3), including the GAI DELLA-type transcriptional regulator, which exhibited an app. 1,5-fold increased expression in the mutant. The expression of other DELLA protein genes showed a slightly but not statistically significant increase in this line. GA-related DEGs also included the AtHB23 homeobox protein that is involved in light-regulated hypocotyl growth and cotyledon expansion [21] and the GA catabolic enzyme GA2ox6 [22] (Table 2 and Supplementary Table S3). GA transport is considered to be mediated by NPF (NRT1/PTR FAMILY) transporters [23,24,25]. The NPF family has 53 members in Arabidopsis [26], but the expression of only 6 of them was altered in the *rlck vi_a2* mutant. Interestingly, four out of these 6 NPFs have already been implicated in GA transport (Table 2 and Supplementary Table S3) [24,25,27]. Expressions of selected GA-related transcripts in the mutant and wild type background were also tested by qRT-PCR (Supplementary Figure S5a). The results supported the validity of the transcriptomic data.

**Figure 7:**
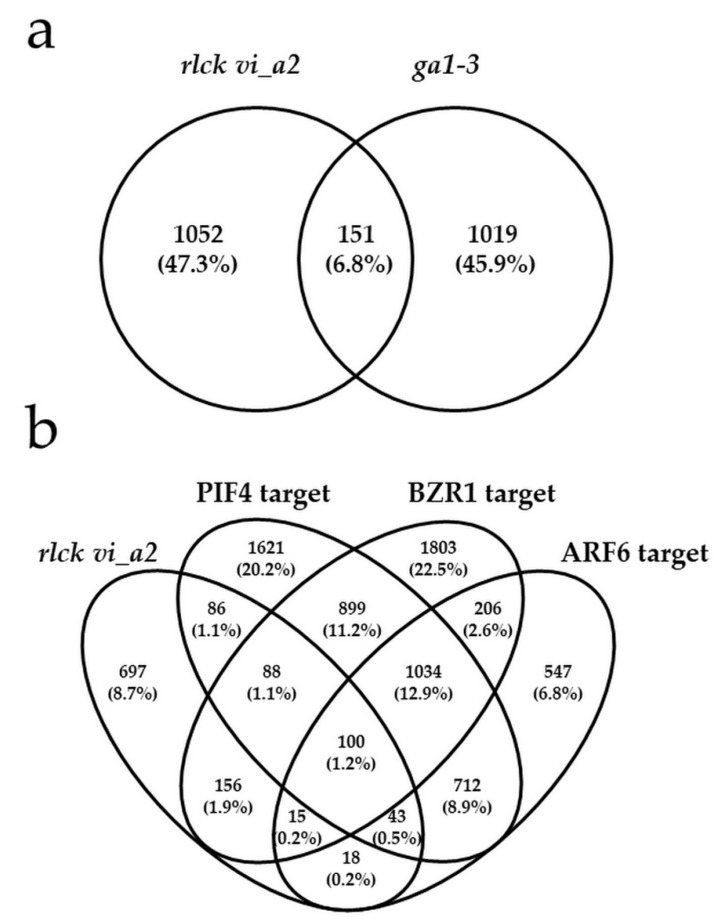
Overlaps of the DEGs of the *rlck vi_a2* mutant with the DEGs of the *ga1-3* gibberellin synthesis mutant [28,29] (**a**) and with those genes for which the promoters are direct targets of the cell/hypocotyl elongation regulatory transcription factors PIF4, BZR1, and/or ARF6 [31] (**b**).

Although only a few of them were directly GA-related, the *RLCK VI_A2*-dependent DEGs showed an overlap (15%), with the DEGs identified in the GA synthesis defective mutant *ga1-3* [28,29] (Figure 7 and Supplementary Table S4) suggesting an indirect and limited effect of the kinase on GA signalling.

Hypocotyl elongation is coordinated by the light and GA-regulated PIF transcription factors [30], as well as by the brassinosteroid- and auxin-controlled transcription factors BZR1 and ARF6, respectively [31]. The transcription factors were shown to interact with each other, forming a central growth regulatory circuit [31]. Promoters of thousands of genes were identified to be direct and partly common targets of the PIF4, BZR1 and ARF6 factors in relation to hypocotyl cell elongation [31]. Among the 1019 DEGs affected by the *rlck vi_a2* mutation, 317 (31%) belong to direct PIF4 targets, 359 (35%) is the direct target of BZR1 and 176 (17%) of ARF6, respectively, and 100 (9.8%) of them bind all three TFs (Figure 7 and Supplementary Table S5). PIF4 itself was found to be slightly upregulated (1.5-fold) in the mutant. Expressions of selected transcripts involved in the regulation of hypocotyl elongation during skotomorphogenesis were also tested in the mutant and the wild type by qRT-PCR (Supplementary Figure S5b). The obtained results supported the validity of the transcriptomic data.

Altogether, these data imply that GA level/signalling is not the primary target of the RLCK VI_A2 kinase, at least not at the gene transcription level.

### 2.7. Transcriptome Analysis Revealed the Role of the RLCK VI_A2 Kinase in Cellular Transport and Cell Wall Organisation

High number of the *RLCK VI_A2*-related DEGs code for proteins with catalytic, transport, transducer or binding activities, respectively (Table 1). Considering cellular localisation, proteins with extracellular (279, 23%) or cell periphery (395, 33%) localisation, and with association to cellular membranes (530, 44%), including the endomembrane system (118, 10%), were significantly overrepresented by the DEGs (Table 1 and Supplementary Tables S2 and S6). Moreover, 6% (72) of the DEGs are implicated in cell wall organisation and biogenesis (Supplementary Tables S2 and S6). Of note is the upregulation of several expansin and xyloglucan endotransglucosylase/hydrolase coding genes and the downregulation of those coding for extensin-like family proteins in the mutant background (Supplementary Table S6; for qRT-PCR validation Supplementary Figure S5c). Eight percent of DEGs (101) code for proteins that have transmembrane transport activities, including ion, nutrient or hormone transporters (Supplementary Table S6). Worth mentioning of the upregulation of several tonoplast intrinsic proteins and a number of auxin transporters, such as PIN4, PIN7, EIR1, ABCB19, LAX2, and the downregulation of many ion transporters (phosphate, sulphate, potassium etc.). These data indicate that RLCK VI_A2-mediated protein phosphorylation is required for the proper cellular transport of a wide variety of ions, nutrients, structural and regulatory molecules.

## 3. Discussion

Plant growth and development have to be continuously harmonized with external conditions. Protein kinases have central roles in sensing environmental signals, as well as in coordinating cellular and developmental responses. Plants possess a large superfamily of diverse protein kinase types, including the signal-sensing transmembrane receptor kinases, RLKs, and various types of downstream intracellular signal-transducing kinases. Among the latter, receptor-like cytoplasmic kinases (RLCKs) have recently gained increasing interest as potential mediators and modulators of RLK signalling [4,32,33]. Nevertheless, only a few of the 149 Arabidopsis and 187 rice RLCKs have been characterized and have known functions. Here, we describe the characterization of a T-DNA insertion mutant of At*RLCK VI_A2* that implies roles for the kinase in the regulation of cell/plant growth and morphogenesis.

### 3.1. The RLCK VI_A Kinases Are Required for Cell Elongation and Organ Growth in Addition to Their Role in Stress Responses

The RLCK VI_A3 kinase of Arabidopsis and its barley homologue, HvRBK1, have been implicated in ROP-GTPase-dependent pathogen resistance/susceptibility reactions [6,7,9], while the AtRLCK VI_A4/AtRBK1 and AtRLCK VI_A6/AtRBK2 kinases were shown to be expressed in response to pathogen infection [7], suggesting their primary role in plant defence. A considerable number of DEGs of the *rlck vi_a2* mutant are also related to plant defence (Table 1), strengthening this view. In addition, the transcript analysis indicated that the kinase might function during abiotic stress responses as well (Table 1). However, the involvement of several RLCK VI_A kinases in the regulation of plant growth and development has also been reported: the T-DNA mutant of the related *AtRLCK VI_A3* kinase is stunted and has over-branched trichomes [9]; the Arabidopsis RBK1 protein kinase (RLCK VI_A4) has been implicated in auxin-responsive cell expansion, due to the reduced auxin responsiveness of its T-DNA insertion mutant [14].

Our results show that the mutant seedlings producing no full length RLCK VI_A2 kinase (Figure 1) have limited cell expansion as compared to the wild type (Figure 3) resulting in shorter hypocotyls and cotyledons (Figure 2). Independent experiments using RNA interference to reduce *RLCK VI_A2* expression (Supplementary Figure S2), as well as the complementation of the T-DNA-caused mutation in transgenic lines (Figure 2) confirmed that the observed phenotypes are indeed associated with the absence of the kinase. Greenhouse-grown mutant plants also exhibited smaller rosette/leaf size than the wild type similarly to the reduced plant size of the related AtRLCK VI_A3 kinase [9].

Altogether, these observations strengthen the view that RLCK VI_A members have a general role in cell expansion and plant growth. Interestingly, while mutation in *AtRBK1/AtRLCK VI_A4* resulted in increased auxin sensitivity, in our experiments, the *atrlck vi_a2* mutant showed no altered auxin response (Supplementary Figure S3), but the mutant phenotypes could be rescued by exogenous GA_3_ (Figure 5 and Supplementary Figure S4). This indicates that the various RLCK VI_A kinases might influence cell expansion via various pathways.

### 3.2. How the RLCK VI_A2 Kinase May Affect Cell Expansion?

How RLCK VI_A2 kinase regulate cell expansion is not known at present. It has to be mentioned that the barley HvRBK1/HvRLCK VI_A3 kinase has been shown to be required for proper cortical microtubule organisation; the silencing of HvRBK1 was shown to result in a fragmented cortical microtubule network [6]. Since cortical microtubules are known to control directional cell elongation [34,35] in a ROP GTPase-dependent manner [36], the ROP-binding kinases might be involved in this process. Transcriptome analysis during the skotomorphogenesis of *rlck vi_a2* mutant seedlings indicates that high portion of the DEGs modulated in the mutant code for proteins located at membranes, at the cell periphery or in the apoplast, and may have a role in transport or cell wall organisation (Table 1 and Supplementary Tables S2 and S6). Therefore, it is conceivable that the RLCK VI_A2 kinase has a regulatory role in these processes in relation to cell elongation. The transcriptomic data, however, do not provide a clear view about the role of the kinase in cell elongation. The upregulation of several expansins and xyloglucan endotransglucosylase/hydrolase coding genes implicated in cell wall loosening [37,38] and the downregulation of those coding for extensin-like family proteins rather contributing to cell wall stiffening [39,40] are not consistent with the observed phenotype of the mutant having restricted cell elongation. Moreover, PIF4, a positive regulator of skotomorphogenesis including hypocotyl cell elongation is upregulated in the *rlck vi_a2* mutant, despite its short-hypocotyl phenotype [30]. These contradictions might be resolved by keeping in mind that the kinase primarily modulates posttranslational and not transcriptional regulation. The observed transcriptional changes might be indirect responses to the missing kinase function: blocking cell elongation at the posttranslational level (e.g., phosphorylation-dependent degradation of PIF4 or other regulators) might give a feedback to increase the transcription of genes promoting cell expansion. For example, PIF4 has been shown to be phosphorylated by the brassinosteroid signalling kinase BRASSINOSTEROIDINSENSITIVE 2 (BIN2), marking it for proteasomal degradation [41]. Considering the number of genes affected by the *rlck vi_a2* mutation, and being at the same time the direct targets of the PIF4/BZR1/ARF6 transcriptional factor circuit that centrally controls cell expansion and hypocotyl growth (Figure 7 and Supplementary Table S5), one can suppose that the kinase directly and/or indirectly modulates the downstream processes controlled by these factors.

### 3.3. Gibberellin might Indirectly Complement for the Missing Kinase Function

Although exogenous GA_3_ treatment could rectify the absence of RLCK VI_A2 function (Figure 5 and Supplementary Figure S4), the kinase mutant exhibited similar bioactive GA levels than the wild type (Figure 6a). Moreover, there was no decrease in the GA sensitivity of the mutant (Figure 6b) despite a ca. 1.5-fold increase in the expression of the GRAS-domain GAI protein gene (Table 2, Supplementary Figures S3 and S5a), a negative regulator of GA signalling. Transcriptomic analysis confirmed that genes implicated in gibberellin metabolism are hardly affected by *RLCK VI_A2* expression. However, the same analysis indicated the misregulation of 15% of *RLCK VI_A2*-dependent DEGs also in the GA synthesis defective mutant *ga1-3* [28,29] (Figure 7a and Supplementary Table S4). Furthermore, the RLCK VI_A2 kinase might be involved in the modulation of GA transport, since the expression of four potential GA transporters [25,26,28] was found to be regulated in the mutant background (Table 2).

The above observations indicate that, although RLCK VI_A2 functions interfere with GA action, this is likely not through the direct modulation of the synthesis of bioactive GAs. Transcriptomic analysis revealed that the absence of the RLCK VI_A2 kinase affected the signalling of auxin and BR, the two other hormones also centrally involved in the light and developmental regulation of hypocotyl elongation [31]. Both the auxin and BR hormones are well known to crosstalk with GA modulating each other’s metabolism and signalling [37,38,39]. Although all three hormones (GA, BR and auxin) act on distinct TFs governing hypocotyl elongation (PIF4, ARF6, and BZR1, respectively), the target genes of these transcription factors largely overlap [31] (Figure 7b). A considerable fraction of the DEGs of the *rlck vi_a2* mutant (31.6%) are direct targets of at least one of the above TFs, while 10% of the DEGs is direct target of all three. The observed gene expression changes might be indirect consequences of blocked cell expansion/seedling growth. The data support the view that the RLCK VI_A2 kinase might control basic cell elongation processes downstream of the PIF4/BZR1/ARF6 TFs, rather than the regulatory proteins themselves.

Why exogenous GA_3_, but not auxin or brassinosteroid, rescue the *rlckvi_a2* mutant phenotypes is not known. Exogenous GA_3_ might induce parallel pathways that can overcome the cell elongation defects caused by missing protein phosphorylations in the absence of the RLCK VI_A2 kinase (e.g., via other kinases and/or microtubule/protein stability and/or cell wall organisation, etc.). One of such shared pathways is the regulation of auxin transport, since several auxin transport protein genes are regulated in the mutant background (Supplementary Table S3), and GA is known to affect auxin transport stabilizing these proteins [42].

## 4. Materials and Methods

### 4.1. Plant Material and Growth Conditions

Seeds of *Arabidopsis thaliana* (L.) Columbia-0 and mutant lines of the GABI-Kat Arabidopsis T-DNA insertional mutant collection [15], GABI_435H03 and GABI_676D12, were obtained from the Nottingham Arabidopsis Stock Centre (NASC).

The full-length cDNA of At2G18890 was obtained from the Arabidopsis Biological Resource Centre (ABRC, Columbus, OH, USA; stock number U67191). The cDNA was amplified (denaturation 94 °C 10 s, annealing temperature 62 °C for 30 s, elongation 1 min at 72 °C) by the proof-reading PHUSION™ II polymerase (Thermo Fisher Scientific, Waltham, MA, USA), using specific primers, having added EcoRI and XhoI sites (Supplementary Table S7). The gel-purified PCR fragment was digested by FastDigest™ EcoRI and XhoI enzymes (Thermo Fisher Scientific) and inserted into similarly cut and purified pENTR2B vector (Thermo Fisher Scientific). The cDNA was cloned into the plant expression vectors pN-TAPa (Gene bank accession: AY788908 [17]) and pMDC7 [18,43] via Gateway recombination, using standard LR Clonase™ II (Thermo Fisher Scientific, catalogue number: 11791019) reaction, as recommended by the supplier. The binary vectors were transformed into GV3101/pMP90 Agrobacterium strain with tri-parental mating [44], which were used for transgenic *Arabidopsis thaliana* (GABI_435H03 and/or Col-0) production via floral dip agroinfiltration [45]. Seeds were selected using appropriate antibiotics (gentamycin and hygromycin, respectively). Plants (including the T-DNA insertion mutants) were characterized for *RLCK VI_A2* (AT2G18890) expression, using the same primers as for cloning in reverse transcription polymerase chain rection (RT-PCR) (see later). Transgenic lines with appropriate expression were selfed and propagated. Stable, homozygous T3/T4 transgenic plants were used in the experiments.

Seeds of the wild type, mutant and transgenic lines were sterilized in 2% bleach, resuspended in sterile water and stratified (4 °C for 48 h). Germination was performed in vertically oriented square Petri dishes with half strength Murashige and Skoog (MS) medium containing 0.5% sucrose 0.8% agar, pH 5.7 (Duchefa Biochemie, Haarlem, The Netherlands). When estradiol inducible lines were used, the growth medium contained 5 µM β estradiol (E2758, Sigma, St. Louis, Mo, USA) for gene expression induction. The experiments were done in growth chambers (Aralab, Rio de Mouro, Portugal) under short days (8 h 22 °C, 120 µE light intensity and 16 h 21 °C darkness); in complete darkness at 22 °C to investigate skotomorphogenesis; or in continuous low white light at 60 µE for the gibberellin sensitivity assay.

### 4.2. Analysis of Hypocotyl Length and Rosette Size Measurement

The hypocotyl and cotyledon lengths were measured from digital photographs by the ImageJ software (NIH, Bethesda, MD, USA). At least 60 wild-type and mutant seedlings were analysed in three biological replicates. For the determination of plant rosette size, 28 or 33-days-old Arabidopsis plants grown in pots were photographed, and rosette diameters were measured with the “straight line” function of the ImageJ software [46]. The distance between the tips of the two longest rosette leaves were measured and exported to MS Excel file for further analysis. Images were taken with a digital camera (Fuji FinePix S1000fd) using the same parameters (focus distance, resolution, ISO).

### 4.3. Cell Size and Shape Analysis

The analysis of hypocotyl cell length was done after Acridine orange (100 μg/mL) staining. Images on the three different part of the hypocotyls (basal, middle, top) were taken by confocal laser scanning microscopy (TCS SP5, Leica Microsystems, Heidelberg, Germany), and the cell length was determined using the ImageJ software [46].

Cotyledon epidermal cell size and shape were visualized uncoated in a JSM-7100F/LV scanning electron microscope (JEOL Ltd., Akishima, Tokyo, Japan) at low-vacuum, by detecting backscattered electrons according to [47]. The images were taken from the same zone of the cotyledons (at the centre, next to the main vein) and perimeter, area, and circularity of cells were analysed using the ImageJ software [46].

Altogether, 150–200 cells were measured per line, in three repetitions.

### 4.4. Gibberellin Content Measurement by Ultra-High Performance Liquid Chromatography-Tandem Mass Spectrometry

The sample preparation and analysis of GAs were performed according to the method described in [48] with some modifications. Briefly, tissue samples of 26–60 mg DW (three independent technical replicates of each of the tree biological samples) were ground to fine consistency using 3-mm zirconium oxide beads (Retsch GmbH & Co. KG, Haan, Germany) and an MM 301 vibration mill at a frequency of 30 Hz for 3 min (Retsch GmbH & Co. KG, Haan, Germany), with 1 mL of ice-cold 80% acetonitrile, containing 5% formic acid as extraction solution. The samples were then extracted overnight at 4 °C using a benchtop laboratory rotator Stuart SB3 (Bibby Scientific Ltd., Staffordshire, UK), after adding 17 internal GA standards ([2H2]GA1, [2H2] GA_3_, [2H2]GA4, [2H2]GA5, [2H2]GA6, [2H2]GA7) purchased from OlChemIm, Olomouc, Czech Republic. The homogenates were centrifuged at 36,670× *g* and 4 °C for 10 min; corresponding supernatants further purified using reversed-phase and mixed mode SPE cartridges (Waters, Milford, MA, USA) and analysed by ultra-high performance liquid chromatography-tandem mass spectrometry (UHPLC-MS/MS; Micromass, Manchester, UK). GAs were detected using multiple-reaction monitoring mode of the transition of the ion [M–H]- to the appropriate product ion. Masslynx 4.1 software (Waters, Milford, MA, USA) was used to analyse the data and the standard isotope dilution method [49] was used to quantify the GAs levels.

### 4.5. Hormone Treatments

For hypocotyl and cotyledon measurements, the plants were germinated in vertically oriented square Petri dishes in 22 °C, under SD conditions or in continuous darkness as indicated. 6-day-old seedlings were moved to 5 nM IAA (I2886 Sigma, St. Louis, MO, USA)-containing medium for additional 6 days before measured. Epibrassinolid (1 μM; E1641 Sigma) or Ethrel (100 μM; Bayer CropSience, Gent, Belgium) or GA_3_ (20 µM; G7645 Sigma) were included into the medium from the beginning of culture. Hormone concentrations not inhibiting hypocotyl elongation were selected based on previous studies [50,51,52,53]. Transgenic RNAi seedlings and corresponding controls were grown in the presence of 5 µM β estradiol in addition to the hormones.

For the gibberellic acid sensitivity assay, constant 60 µE white light was used to limit dark-induced elongation at 22 °C. GA_3_ was included into the growth medium in concentrations indicated on the figure.

For the complementation of the rosette size, 14-day-old, soil-grown plants were sprayed with 20 µM GA_3_ solution supplemented with 0.01% Silwet L-77 (Kwizda, Vienna, Austria). The treatment was repeated at 4-day intervals. The control plants were sprayed with 0.01% Silwet L-77 solution. The GA_3_ was dissolved in DMSO:methanol solution (1:1) and stock solutions were prepared at 1 μΜ (GA_3_) concentrations for further dilution in water. Rosette size was determined as described earlier, 19 days following the start of the treatment (33-day-old plants).

### 4.6. Characterization of Mutant/Transgenic Plants by RT-PCR

The Quick-RNA Plant Miniprep Kit (Zymo Research, Irvine, CA, USA) was used to isolate total RNA from whole seedlings. Total RNA was treated by RNase-free DNase I (Thermo Fisher Scientific) and cDNA templates were generated from 0.5 mg RNA samples by RevertAid M-MuLV reverse transcriptase (Thermo Fisher Scientific). The full length transcript of the *RLCK VI_A2* gene was amplified with primers that were planned for cloning the cDNA in standard PCR reaction (denaturation 94 °C for 30 s, annealing temperature 55 °C for 30 s, elongation 1 min at 72 °C) with DreamTaq polymerase (Thermo Fisher Scientific). *AtGAPC-2* (AT1G13440) transcripts were used as internal reference. See primers in Supplementary Table S7.

Genomic DNA of the GABI_435H03 T-DNA insertion mutant was isolated with the Phire Plant Direct PCR Kit (Thermo Fisher Scientific) and the T-DNA insertion site was amplified with an At2G18890-specific forward primer (VIA2mid_F) and a T-DNA-specific reverse primer (T-DNA LB out), according to the supplier’s instructions. The purified PCR products were sequenced using the same primers. For the primer sequences, see Supplementary Table S7.

### 4.7. RNA-Seq and Data Analysis

The Quick-RNA Plant Miniprep Kit (Zymo Research, Irvine, CA, USA) was used to isolate total RNA from 18-day-old dark-grown seedlings after removing their roots. The RNA preparations were quality checked and quantified using the Agilent RNA 6000 Nano Kit in an Agilent 2100 Bioanalyzer capillary gel electrophoresis instrument (Agilent, Santa Clara, CA, USA). For sequencing library preparation, polyA RNAs were selected from 800 ng total RNA using NEBNext Poly(A) mRNA Magnetic Isolation Module, then strand specific indexed libraries were prepared with NEBNext Ultra II Directional RNA Library Prep Kit for Illumina (New England Biolabs, Ipswich, MA, USA). Libraries were validated and quantified with an Agilent DNA 1000 kit in a 2100 Bioanalyzer instrument, then after pooling and denaturing, library pools were sequenced in an Illumina MiSeq instrument with MiSeq Reagent Kit V3-150 (Illumina Inc., San Diego, CA, USA), generating 2 × 75 bp paired-end reads. Fasq files were trimmed and adapter sequences removed with Trimmomatic 0.33 in paired-end mode [54]. Paired sequences were aligned to the TAIR10 Arabidopsis reference genome using TopHat2 [55]. Binary alignment (*.bam) files were sorted and deduplicated with SAMtools (http://samtools.sourceforge.net/), then differential expression analysis was done with Cufflinks (http://cufflinks.cbcb.umd.edu/), using Araport 11 transcript annotation [56]. Differential expression was considered as significant with a q value lower than 0.05.

The RNA-seq data used for the analysis have been deposited in the NCBI Sequence Read Archive (SRA) (https://www.ncbi.nlm.nih.gov/sra) under the accession PRJNA644816.

### 4.8. Real-Time Quantitative PCR (qRT PCR)

For qRT PCR, total RNA was purified and converted to cDNA as described under 4.6. The oligonucleotide primers are listed in Supplemental Table S7. A few of them have been previously published in [53]. qRT-PCR reactions were performed using an ABI PRISM 7700 sequence detection system (Thermo Fisher Scientific) and the qPCRBIO SyGreen Mix Hi-ROX master mix (PCR Biosystems Ltd., London, UK) using standard protocol (denaturation 95 °C for 10 min, 40 cycles of 95 °C for 10 s, and 62 °C for 60 s). Ct values were analysed using the RQ manager software (Thermo Fisher Scientific), and then exported to Microsoft Excel for further analysis. The ratio of each mRNA relative to the mRNA of the *Arabidopsis thaliana UBIQUITIN EXTENSION PROTEIN 1* gene (*UBQ1*, AT3G52590) was calculated using the 2^−ΔΔCT^ method. *UBQ1* gene expression was uniform in the wild type and mutant background, as shown by the RNA-seq analysis (see in Supplementary Table S1). The average of the three technical repeats of the WT control was used as reference (unit 1) to calculate relative expression for each gene in the mutant background.

### 4.9. Statistical Analysis

Plant culture experiments were carried out in three independent replicates. The number of investigated individuals per replicate is given in each figure legend. Averages with standard errors are shown in the histograms for growth parameters having high and variable sample numbers (e.g., cell, hypocotyl and cotyledon length measurements). In qRT-PCR experiments, two independent biological samples each, with three technical replicates, were amalgamated and analysed together. Student’s *t*-test was used for pairwise statistical comparison of the mutant/treated samples to the corresponding wild type/control ones (* indicates *p*-value < 0.05, ** indicates *p*-value < 0.005).

## 5. Conclusions

Decreased level or absence of the RLCK VI_A2 kinase in transgenic Arabidopsis lines resulted in restricted cell expansion and organ/plant size under short day conditions, as well as in continuous dark (skotomorphogenesis), in seedlings as well as in greenhouse plants, indicating the general role of the kinase in plant growth. Transcriptomic analysis confirmed that the kinase might be involved in the modulation of processes that are associated with cell membranes, and take place at the cell periphery or in the apoplast, such as cellular transport and cell wall organisation. Although exogenous GA_3_ could rescue the mutant phenotypes, hardly any changes in gibberellin metabolism and/or signalling could be observed in the mutant, indicating that the RLCK VI_A2 kinase and gibberellin might act parallel on the same/similar processes. To clarify the exact role of the kinase in cell expansion and its hormonal regulation, the identification of its in vivo substrates is required.

## Figures and Tables

**Table 1:** Gene ontology classification (Plant GOSlim) of the DEGs for 17-days-old dark-grown *rlck vi_a2* mutant seedlings without roots in comparison to wild type.

*GO Term*	*Ontology*	*Description*	*p-Value*	*FDR*
*GO:0050896*	P	response to stimulus	8.6 × 10^−66^	2.7 × 10^−63^
*GO:0006950*	P	response to stress	1.4 × 10^−47^	2.2 × 10^−45^
*GO:0009605*	P	response to external stimulus	1.8 × 10^−36^	2.00 × 10^−34^
*GO:0009607*	P	response to biotic stimulus	1.9 × 10^−29^	1.6 × 10^−27^
*GO:0009628*	P	response to abiotic stimulus	1.2 × 10^−27^	7.4 × 10^−26^
*GO:0051704*	P	multi-organism process	2.2 × 10^−24^	1.2 × 10^−22^
*GO:0009719*	P	response to endogenous stimulus	8.1 × 10^−24^	3.7 × 10^−22^
*GO:0019748*	P	secondary metabolic process	2.6 × 10^−20^	1.00 × 10^−18^
*GO:0007154*	P	cell communication	3.7 × 10^−15^	1.3 × 10^−13^
*GO:0009056*	P	catabolic process	6.3 × 10^−12^	2.00 × 10^−10^
*GO:0007165*	P	signal transduction	2.8 × 10^−11^	8.3 × 10^−10^
*GO:0009987*	P	cellular process	2.7 × 10^−10^	7.2 × 10^−9^
*GO:0008152*	P	metabolic process	2.2 × 10^−9^	5.4 × 10^−8^
*GO:0051179*	P	localization	3.3 × 10^−9^	7.6 × 10^−8^
*GO:0009991*	P	response to extracellular stimulus	1.5 × 10^−8^	3.1 × 10^−7^
*GO:0006629*	P	lipid metabolic process	2.1 × 10^−8^	4.1 × 10^−7^
*GO:0006810*	P	transport	3.2 × 10^−8^	6.1 × 10^−7^
*GO:0051234*	P	establishment of localization	4.7 × 10^−8^	8.4 × 10^−7^
*GO:0005975*	P	carbohydrate metabolic process	2.2 × 10^−7^	3.7 × 10^−6^
*GO:0008219*	P	cell death	7.9 × 10^−6^	0.00013
*GO:0065008*	P	regulation of biological quality	2.00 × 10^−5^	0.00031
*GO:0065007*	P	biological regulation	2.7 × 10^−5^	0.00039
*GO:0042592*	P	homeostatic process	5.6 × 10^−5^	0.00078
*GO:0040007*	P	growth	0.0009	0.012
*GO:0009606*	P	tropism	0.0011	0.014
*GO:0032502*	P	developmental process	0.0017	0.021
*GO:0050789*	P	regulation of biological process	0.002	0.024
*GO:0050794*	P	regulation of cellular process	0.0025	0.029
*GO:0048856*	P	anatomical structure development	0.0038	0.042
*GO:0003824*	F	catalytic activity	1.3 × 10^−25^	1.3 × 10^−23^
*GO:0005215*	F	transporter activity	4.2 × 10^−9^	2.1 × 10^−7^
*GO:0016740*	F	transferase activity	1.9 × 10^−8^	6.4 × 10^−7^
*GO:0008289*	F	lipid binding	1.2 × 10^−7^	3.00 × 10^−6^
*GO:0030246*	F	carbohydrate binding	5.7 × 10^−6^	0.00012
*GO:0016787*	F	hydrolase activity	1.2 × 10^−5^	0.0002
*GO:0019825*	F	oxygen binding	1.9 × 10^−5^	0.00027
*GO:0016301*	F	kinase activity	2.5 × 10^−5^	0.00032
*GO:0000166*	F	nucleotide binding	0.00014	0.0016
*GO:0005488*	F	binding	0.00021	0.0021
*GO:0060089*	F	molecular transducer activity	0.00041	0.0034
*GO:0004872*	F	receptor activity	0.00041	0.0034
*GO:0016772*	F	transferase activity, transferring phosphorus-containing groups	0.0012	0.009
*GO:0005576*	C	extracellular region	8.6 × 10^−37^	1.6 × 10^−34^
*GO:0030312*	C	external encapsulating structure	6.7 × 10^−34^	4.2 × 10^−32^
*GO:0005618*	C	cell wall	6.7 × 10^−34^	4.2 × 10^−32^
*GO:0005886*	C	plasma membrane	7.5 × 10^−32^	3.5 × 10^−30^
*GO:0016020*	C	membrane	1.3 × 10^−25^	4.9 × 10^−24^
*GO:0005773*	C	vacuole	4.1 × 10^−5^	0.0013
*GO:0005783*	C	endoplasmic reticulum	8.8 × 10^−5^	0.0023
*GO:0012505*	C	endomembrane system	0.0018	0.043

FDR—false discovery rate; P—biological process; F—molecular function; C—cellular compartment.

**Table 2:** Manually identified DEGs implicated in gibberellin metabolism/response/transport in dark-grown 17-days-old *rlck vi_a2* mutant seedlings (see Supplementary Table S3 for more details).

Gene_ID	Annotation	Fold Change	*q*_Value	Significant
DEGs Implicated in GA Metabolism/Response *				
AT1G26960	AtHB23 homeobox protein 23	2.25	0.026242	yes
AT1G74670	GASA6 Gibberellin-regulated family protein	2.07	0.001941	yes
AT2G37640	EXP3 Barwin-like endoglucanases superfamily protein	1.95	0.001941	yes
AT5G15230	GASA4 GAST1 protein homolog 4	1.88	0.001941	yes
AT2G14900	AT2G14900 Gibberellin-regulated family protein	1.70	0.001941	yes
AT1G14920	GAI GRAS family transcription factor family protein	1.46	0.00859	yes
AT3G11280	AT3G11280 Duplicated homeodomain-like superfamily protein	0.69	0.00859	yes
AT4G19700	RING SBP (S-ribonuclease binding protein) family protein	0.68	0.038936	yes
AT1G75750	GASA1 GAST1 protein homolog 1	0.56	0.003506	yes
AT5G44610	MAP18 microtubule-associated protein 18	0.55	0.001941	yes
AT1G02400	GA2OX6 gibberellin 2-oxidase 6	0.58	0.044627	yes
DEGs of NPF (NRT1/PTR FAMILY) Gibberellin Transporters **				
AT1G52190	AT1G52190 Major facilitator superfamily protein, NPF1.2 ^a,b^	1.60	0.001941	yes
AT3G16180	AT3G16180 Major facilitator superfamily protein, NPF 1.1 ^a^	1.79	0.001941	yes
AT5G46050	PTR3 peptide transporter 3, NPF 5.2 ^a^	0.55	0.001941	yes
AT5G62680	GTR2 Major facilitator superfamily protein, NPF 2.11 ^c^	0.65	0.031474	yes
DEGs of DELLA Transcription Regulators ***				
AT1G14920	GAI GRAS family transcription factor family protein	1.46	0.00859	yes
AT2G01570	RGL1 RGA-like 1	1.25	0.483175	no
AT1G66350	RGA1 GRAS family transcription factor family protein	1.11	0.773976	no
AT3G03450	RGL2 RGA-like 2	1.58	0.496428	no
AT5G17490	RGL3 RGA-like protein 3	1.21	0.819669	no

* Manually selected based on the presence of the word “gibberellin” in the annotations of DEGs implicated in “hormone response” by the AgriGO v2.0 tool. ** NPF transporters that were reported to transport gibberellins by ^a^ [24]; ^b^ [27]; ^c^ [25]. *** Note that only the differential expression of *GAI* was statistically significant.

## Supplementary Materials

Supplementary materials can be found at https://www.mdpi.com/1422-0067/21/19/7266/s1. Figure S1: Mapping of transcript sequence reads to the genomic sequence of the At2G18890 gene, Figure S2: Estradiol-induced silencing of the *RLCK VI_A2* gene, Figure S3: Exogenous hormone treatments, Figure S4: GA_3_ complements for the silencing of the *RLCK VI_A2* gene, Figure S5: qRT-PCR validation of the expression of selected genes, Table S1: Full transcriptomic analysis, Table S2: GO enrichment, Table S3: Hormone-responsive DEGs, Table S4: DEGs overlaping with the DEGs of the *ga1*-3 mutant, Table S5: DEGs directly regulated by the hypocotyl elongation controlling TFs, Table S6: DEGs related to cell wall-related and transport processes, Table S7: Sequences of the used oligonucleotide primers.

## Abbreviations

ABCB	ATP-binding cassette B protein
ARF	auxin response factor
AtHB	*Arabidopsis thaliana* homeobox-leucine zipper protein
AtRBK	*Arabidopsis thaliana* ROP-binding kinase
BZR	brassinazole-resistant
DEG	differentially expressed gene
DELLA	protein domain of the key negative regulators of GA action (DELLA proteins)
EIR	ethylene insensitivity of the root
FDR	false discovery rate
GA	gibberellin
ga1	gibberellic acid 1-gibberellin synthesis mutant
GAI	gibberellic acid-insensitive protein
GAPC-2	glyceraldehyde 3-phosphate dehydrogenase C-2
GO	gene ontology
GRAS	GA INSENSITIVE (GAI), REPRESSOR of ga1-3 (RGA), and SCARECROW (SCR) protein domain
GTP	guanosine-5’-triphosphate
HvRBK	Hordeum vulgare ROP-binding kinase
LAX	Like auxin1
MtRRK1	*Medicago truncatula* ROP-activated Receptor-like Kinase 1
NPF	NRT1/PTR family
NRT	nitrate transporter
PCR	polymerase chain reaction
PIF	phytochrome interacting factor
PIN	PINNOID protein
PTR	peptide transporter
RLCK	receptor-like cytoplasmic kinase
RLK	receptor-like kinase
ROP	Rho-of-plants
SD	short day
T-DNA	transfer-DNA
TF	transcription factor
