# Supplementary material for: The Arabidopsis RLCK VI_A2 Kinase Controls Seedling and Plant Growth in Parallel with Gibberellin
Source: Int J Mol Sci. 2020 Oct 1;21(19):7266. doi: 10.3390/ijms21197266 (PMC7582978; doi:10.3390/ijms21197266)
Supplement: Supplementary file 1 [file ijms-21-07266-s001.zip › Supplementary Valkai et al/Figs/Supplementary Fig. 5 sm2.pdf]

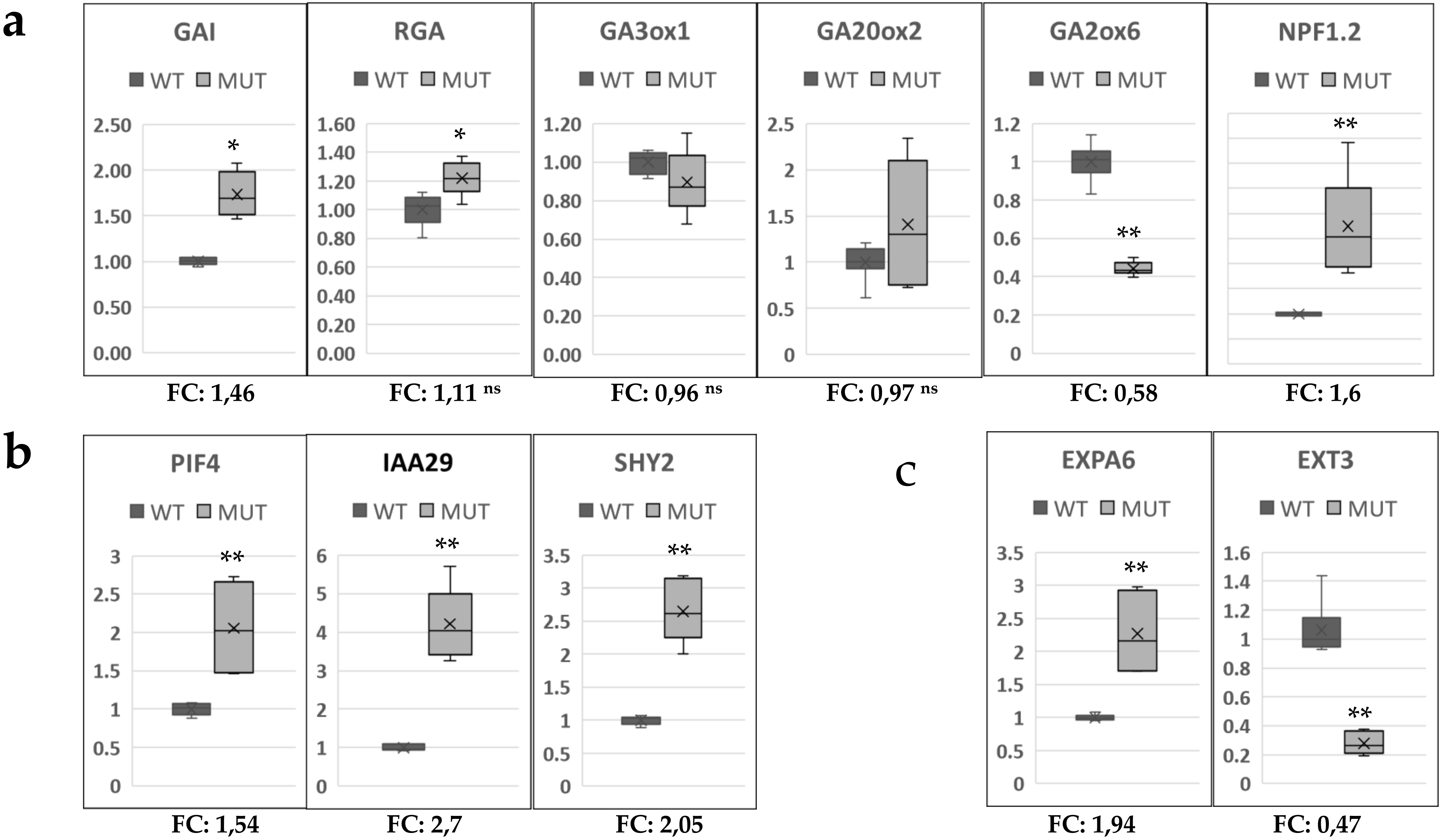

Suppl. Fig. 5. qRT-PCR validation of the expression of selected genes ((**a**) gibberellin metabolism and transport; (**b**) hypocotyl elongation; (**c**) cell wall remodelling) in 18-days-old dark-grown seedlings (without roots). Relative expression data from two biologically indepependent experiments with three technical repeats each are shown together as box plots.
* p<0.05 ** p<0.05 (Student's t-test; comparison to WT). Below the histograms the NGS analysis values are given for the same genes (FC – fold change; ns – not significant with q<0.05)
