# Supplementary material for: The Arabidopsis RLCK VI_A2 Kinase Controls Seedling and Plant Growth in Parallel with Gibberellin
Source: Int J Mol Sci. 2020 Oct 1;21(19):7266. doi: 10.3390/ijms21197266 (PMC7582978; doi:10.3390/ijms21197266)
Supplement: Supplementary file 1 [file ijms-21-07266-s001.zip › Supplementary Valkai et al/Figs/Supplementary Fig.1 sm2.pdf]

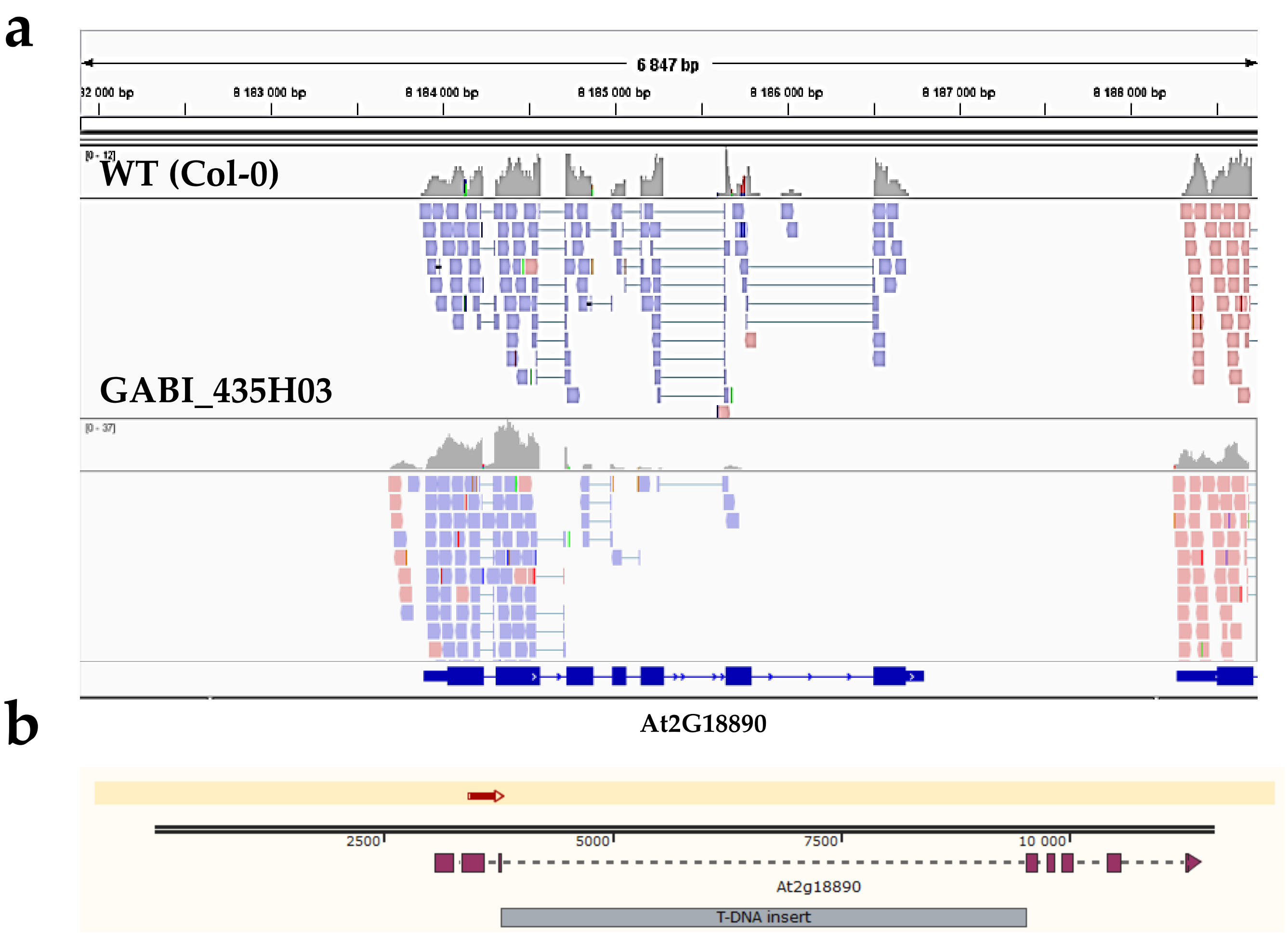

Suppl. Fig. 1. Mapping of transcript sequence reads to the genomic sequence (TAIR10) of the At2G18890 gene coding for the AtRLCK VI_A2 kinase. In the T-DNA insertion mutant, GABI_435H03, only the first two exons of the At2G18890 (*RLCK VI_A2*) gene are transcribed (**a**) in agreement with the site of T-DNA insertion right after the start of the third exon (**b**).
