# Supplementary material for: The Arabidopsis RLCK VI_A2 Kinase Controls Seedling and Plant Growth in Parallel with Gibberellin
Source: Int J Mol Sci. 2020 Oct 1;21(19):7266. doi: 10.3390/ijms21197266 (PMC7582978; doi:10.3390/ijms21197266)
Supplement: Supplementary file 1 [file ijms-21-07266-s001.zip › Supplementary Valkai et al/Figs/Supplementary Fig.2 sm2.pdf]

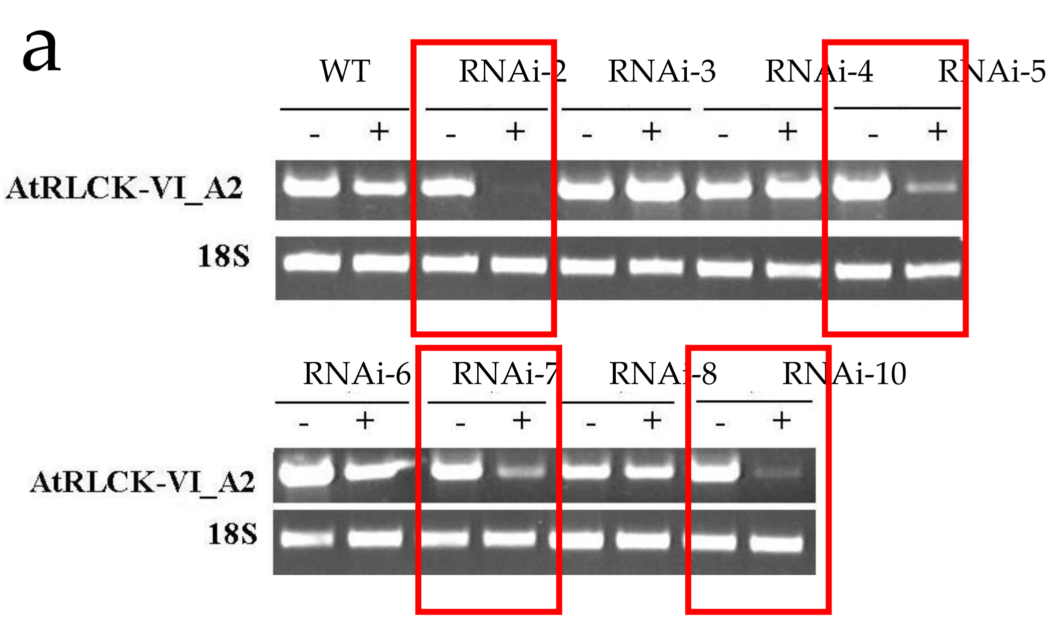

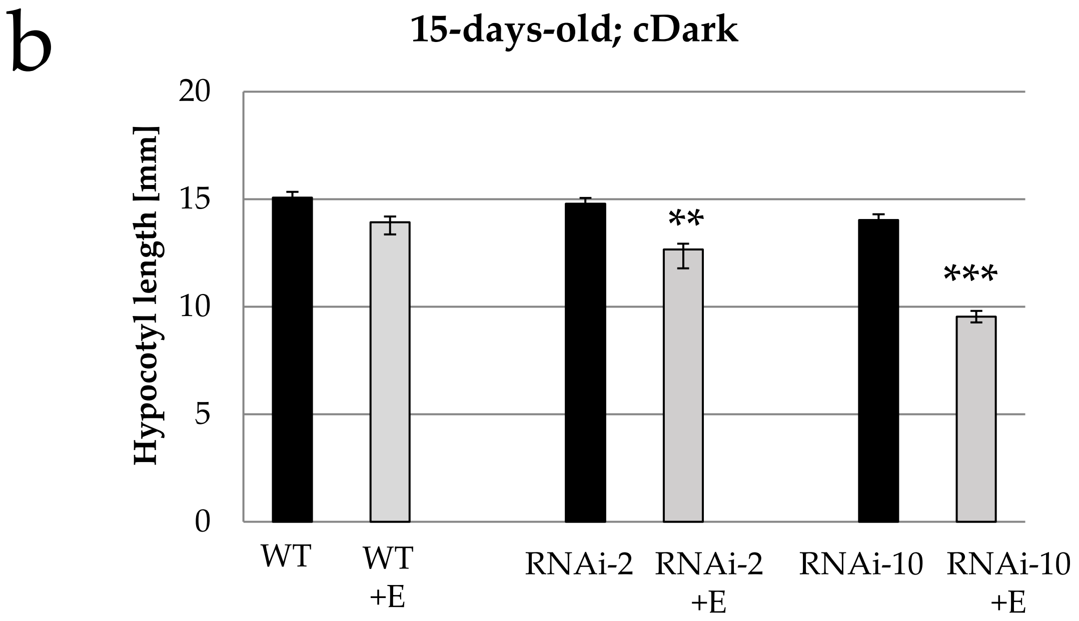

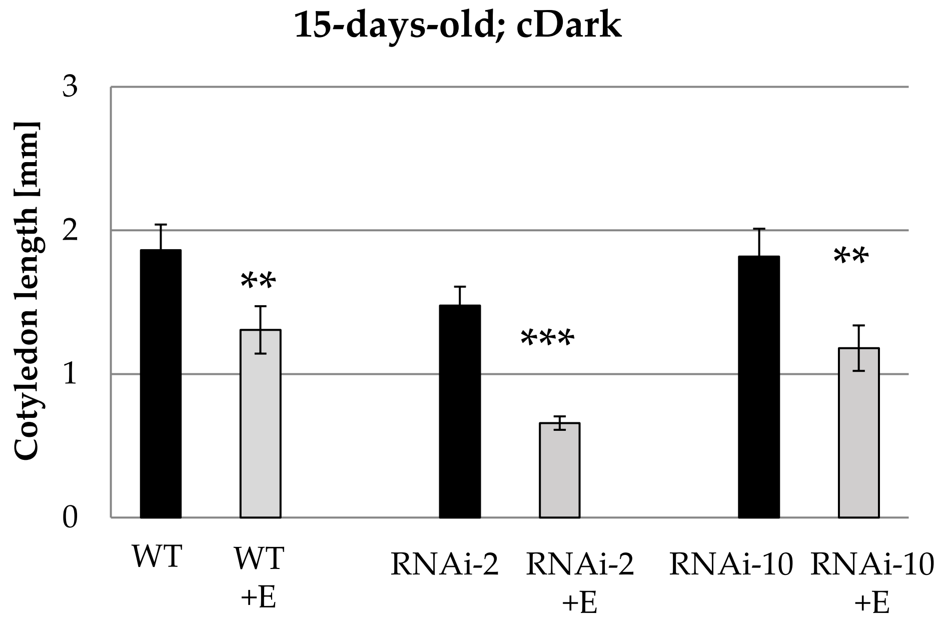

Suppl. Fig. 2. Reproduction of the hypocotyl and cotyledon length phenotype of the T-DNA insertion *rlck vi_a2* mutant via estradiol-induced silencing of the *RLCK VI_A2* gene using RNA interference (RNAi).

(**a**) RT-PCR analysis of the expression of the *RLCK VI_A2* gene without (-) and with (+) estradiol treatment. *18S* ribosomal RNA gene expression was used as control. Transgenic lines with efficient gene silencing are boxed.

WT=wild type Col-0. RNAi-# = independent transgenic lines carrying estradiol-inducible *RLCK VI_A2* transgene (cDNA) in „hairpin" configuration.

(**b**) Hypocotyl and cotyledon length of wild type and transgenic dark-grown (cDark) seedlings (15-days) without and with (+E) estradiol treatment.

Averages and standard errors are shown. Statistically significant differences (t –test) in comparison to the corresponding untreated seedlings (n=20) are indicated: ** $p<0.05$; *** $p<0.01$.
