# Supplementary material for: The Arabidopsis RLCK VI_A2 Kinase Controls Seedling and Plant Growth in Parallel with Gibberellin
Source: Int J Mol Sci. 2020 Oct 1;21(19):7266. doi: 10.3390/ijms21197266 (PMC7582978; doi:10.3390/ijms21197266)
Supplement: Supplementary file 1 [file ijms-21-07266-s001.zip › Supplementary Valkai et al/Figs/Supplementary Fig.3 sm2.pdf]

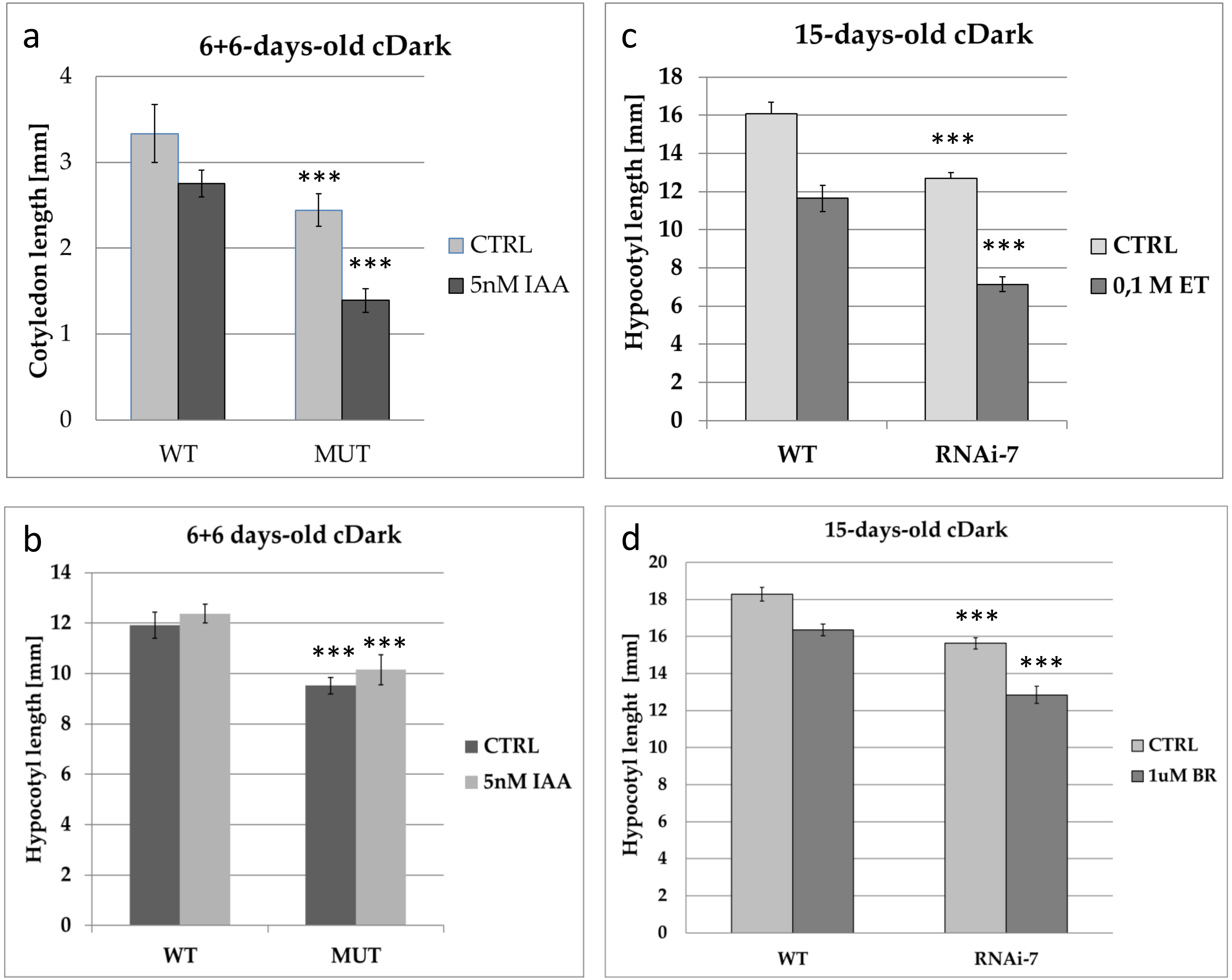

Suppl. Fig. 3. Effect of exogenous auxin (indoleacetic acid, IAA, a-b), brassinolide (BR, c) or the ethylene-releasing Ethrel (ET, d) on the cotyledon and/or hypocotyl size of transgenic plants with no or limited *RLCK VI_A2* expression. 5 nM IAA was applied to 6-day-old dark-grown seedlings for additional 6 days before measured (a-b). BR and ET was present in the medium for 15 days in continuous darkness. WT- wild-type; MUT – T-DNA insertion mutant (see Suppl. Fig. 1); RNAi-7 – transgenic plant with conditionally silenced *RLCK VI_A2* gene. RNAi-7 plants and corresponding controls were grown in the presence of 5 µM β estradiol (see Suppl. Fig. 2). Averages and standard errors are shown. Statistically significant differences (t –test) in comparison to the corresponding control seedlings (n=20) are indicated: *** p<0.01
