# Supplementary material for: The Arabidopsis RLCK VI_A2 Kinase Controls Seedling and Plant Growth in Parallel with Gibberellin
Source: Int J Mol Sci. 2020 Oct 1;21(19):7266. doi: 10.3390/ijms21197266 (PMC7582978; doi:10.3390/ijms21197266)
Supplement: Supplementary file 1 [file ijms-21-07266-s001.zip › Supplementary Valkai et al/Figs/Supplementary Fig.4 sm2.pdf]

Complementation of the cotyledon length defect of dark-grown 17-days-old seedlings having RNAi-silenced *RLCK VI_A2* gene by the application of exogenous gibberellic acid

a

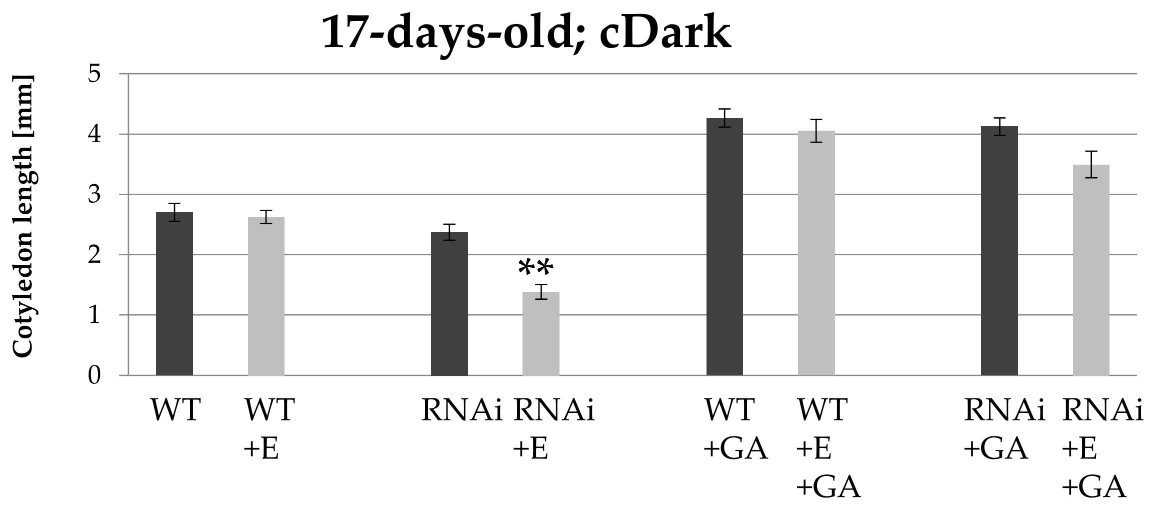

b

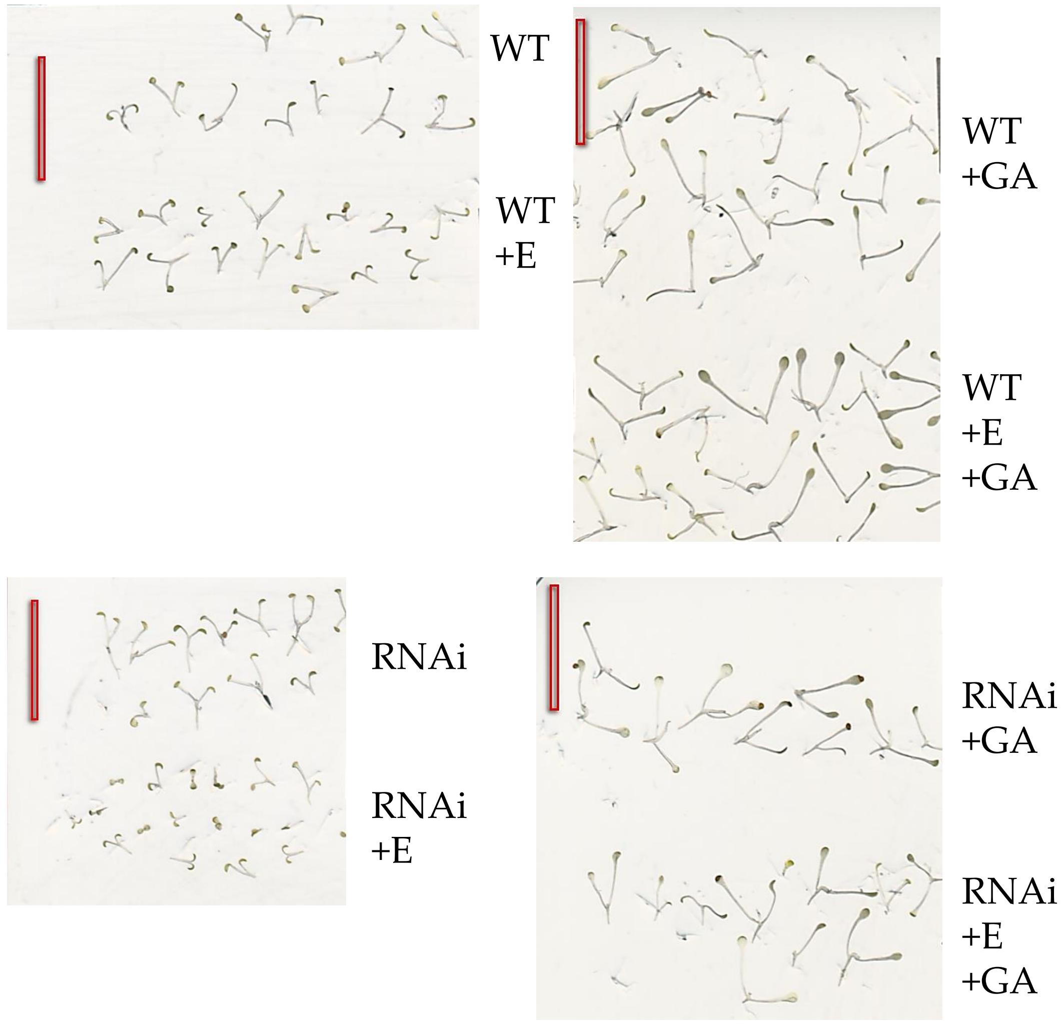

Suppl. Fig. 4. Comparison of the cotyledon length of dark-grown seedlings of the wild-type (WT) and a transgenic Arabidopsis line carrying an estradiol-inducible hairpin RNA interference construct (RNAi-7) to silence the *RLCK VI_A2* gene.
(**a**) Cotyledon length data of control and estradiol (+E) or estradiol and 20 mM GA (+E+GA) treated seedlings.
n>10, averages are shown with standard errors.
** designates statistically significant difference of the RNAi line from the corresponding wild type (p<0,01, t-test).
(**b**) Representative pictures of dissected apical seedling parts used for the measurement. Red bars are 10 mm in length.
